# Relationship Between Protein Intake in Each Traditional Meal and Physical Activity: Cross-sectional Study

**DOI:** 10.2196/35898

**Published:** 2022-07-12

**Authors:** Takae Shinto, Saneyuki Makino, Yu Tahara, Lyie Nitta, Mai Kuwahara, Ayako Tada, Nanako Abe, Mikiko Michie, Shigenobu Shibata

**Affiliations:** 1 Department of Bioscience and Engineering Waseda University Tokyo Japan; 2 Asken Inc Tokyo Japan

**Keywords:** protein, dietary pattern, physical activity, chrononutrition

## Abstract

**Background:**

Protein intake plays an important role in the synthesis and maintenance of skeletal muscles for the prevention of health risks. It is also widely known that physical activity influences muscle function. However, no large-scale studies have examined the relationship between daily dietary habits, especially the timing of protein intake, and daily physical activity.

**Objective:**

The purpose of this cross-sectional study was to investigate how protein intake and composition (involving the 3 major nutrients protein, fat, and carbohydrate) in the 3 traditional meals (breakfast, lunch, and dinner) are associated with physical activity.

**Methods:**

Using daily dietary data accumulated in the smartphone food log app “Asken” and a web-based cross-sectional survey involving Asken users (N=8458), we analyzed nutrient intake and composition, as well as daily activity levels. As very few individuals skipped breakfast (1102/19,319 responses, 5.7%), we analyzed data for 3 meals per day.

**Results:**

Spearman rank correlation analysis revealed that breakfast and lunch protein intakes had higher positive correlations with daily physical activity among the 3 major macronutrients (*P*<.001). These findings were confirmed by multivariate logistic regression analysis with confounding factors. Moreover, participants with higher protein intake and composition at breakfast or lunch tended to exhibit significantly greater physical activity than those with higher protein intake at dinner (*P*<.001).

**Conclusions:**

Among the 3 macronutrients, protein intake during breakfast and lunch was closely associated with daily physical activity.

## Introduction

Maintaining and increasing muscle mass are critical for preventing various health risks, such as metabolic syndrome, diabetes, and sarcopenia [1-3]. In addition, protein intake is an effective factor for skeletal muscle synthesis and maintenance [4]. According to the Ministry of Health, Labor, and Welfare in Japan, the recommended daily protein intake values for adult Japanese males and females are 65 g and 50 g, respectively [5]. Additionally, several studies in the United States and Japan have shown that average protein intake in adults was unevenly distributed across the 3 meals, with the lowest intake at breakfast and the highest intake skewed toward dinner [6,7]. This typical pattern of protein intake has been associated with a decline in muscle function, including grip strength and muscle mass [8-10]. A cross-over study reported significantly higher 24-h muscle protein synthesis rates when protein intake was evenly distributed with adequate consumption at each meal, rather than skewed toward the evening meal [11]. In particular, breakfast is the most commonly skipped meal of the day, yet it plays an important role in health [12-14]. Indeed, recent studies have shown that breakfast protein intake effectively induced muscle hypertrophy in humans and rodents [15,16]. Therefore, it is important to consider not only the total protein intake, but also the intake and composition at each meal for regulating muscle function. In addition, physical activities, such as exercise and strength training, are effective for maintaining and increasing muscle mass [17]. However, the precise relationship between daily eating habits, especially protein intake patterns, and physical activity remains unclear. Therefore, we hypothesized that breakfast protein would have a strong positive relationship with physical activity among nutrients or intake timing. We investigated the relationship between the intake and composition of nutrients (protein, fat, and carbohydrate) in 3 meals and physical activity in approximately 8000 users of the Asken mobile health app for dietary management. Since this is a health care app generally aimed at weight maintenance and weight loss, the users are likely to be highly health conscious. In fact, the percentage of those who skip breakfast in Japan is 12% [18], while in this study, it was as low as 5.7%. In addition, most people consume snacks, but due to data availability, the analysis in this survey focused on the 3 traditional meals. To our knowledge, no previous study has investigated the dietary pattern of 3 meals and physical activity in such a large population. Therefore, this study could potentially clarify the characteristics of physical activity in the daily dietary pattern of 3 meals, including breakfast, as well as the relationship between physical activity and protein intake.

## Methods

### Study Participants and the Mobile Health App “Asken”

This study was approved by the Ethics Review Committee on Research with Human Subjects at Waseda University (No. 2020-046), in accordance with the guidelines of the Declaration of Helsinki. Moreover, informed consent was obtained from all individuals who participated in the study.

**Figure 1:**
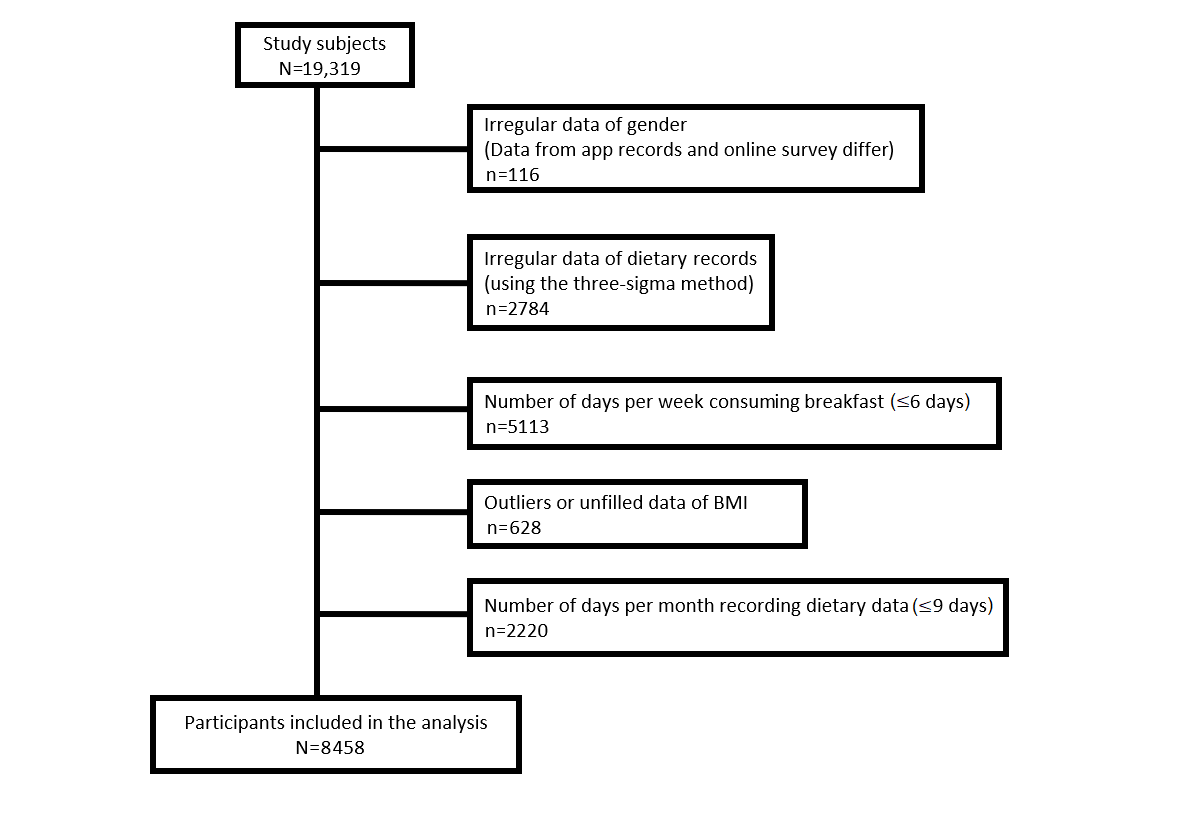
Flow of the study participants.

“Asken” is a popular food log and food coaching app that has been downloaded over 6,000,000 times [19] and has persistently ranked in the top 3 for health app categories in Japan [20]. As most users (almost 95%) use this app for body weight reduction and female individuals might be more inclined to maintain body shape than male individuals, 70% of the app users were female. The app provides feedback on dietary content based on the Dietary Intake Standards for Japanese as determined by the Ministry of Health, Labor, and Welfare. In this study, an online survey was conducted via the app, in addition to the available dietary records. The online survey was conducted in January 2021, and at the time of implementation, we announced that 200 people would be given 500 Japanese yen (approximately US $5) in a lottery. Since the app was not for clinical use and we did not ask for detailed information (eg, disease) from the participants, inclusion and exclusion criteria were not set when the online survey was conducted. The study participants included 8458 users of Asken (aged 10-80 years). As shown in Figure 1, we excluded those who gave contradictory or no responses for gender and BMI, those who did not consume breakfast daily, those whose dietary records were less than 9 days per month, and those who deviated from the normal distribution for dietary intake (using the 3-sigma method).

The self-reported food logs accumulated in the app have been deemed reliable for research purposes [21]. A recent study reported that the median correlation coefficient between daily paper-based nutrient intakes and those obtained from Asken was approximately 0.8 in adult men and women [22]. In addition, the dietary data obtained in this study were comparable with data calculated from the National Nutrition Survey in Japan (NNSJ) [23] (Multimedia Appendix 1). These results suggest that the dietary data used in this study are reliable. However, the amount of daily protein was slightly higher, while the amounts of fat and carbohydrate were slightly lower compared with the NNSJ data (Multimedia Appendix 1).

### Questionnaire

The online survey included 34 items involving personal characteristics (age, gender, and prefecture of residence), lifestyle habits like diet and sleep, physical activity (International Physical Activity Questionnaire [IPAQ] short form), and personality assessment (Ten-Item Personality Inventory [TIPI]). Except for the physical activity and personality assessment, the other items were originally developed by us. In this study, “age,” “gender,” “physical activity (IPAQ short form),” and “frequency of breakfast intake” were used for analysis in the online survey. The questionnaire was designed to take approximately 15 minutes to complete.

### Dietary Data

The dietary assessment was based on the average dietary records of each participant for approximately 1 month (January 2021). For each of the 3 traditional meals (breakfast, lunch, and dinner) and snacks, the intake included energy content (kcal), protein, fat, carbohydrate, sodium, potassium, cholesterol, fiber, and saturated lipids. Herein, the 3 major nutrients (ie, carbohydrate, fat, and protein) were evaluated. As timing information on snacks was lacking, data on snacks were not used for further analysis. To evaluate the compositions of the 3 macronutrients (% kcal), we converted the amount of intake (g) into the amount of energy. The calculation formulas are as follows:

Protein composition (% kcal) = protein intake (g) × 4 (kcal/g) / total energy (kcal) × 100 **(1)**

Fat composition (% kcal) = fat intake (g) × 9 (kcal/g) / total energy (kcal) × 100 **(2)**

Carbohydrate composition (% kcal) = carbohydrate intake (g) × 4 (kcal/g) / total energy (kcal) × 100 **(3)**

In addition, to emphasize the relationship between protein and physical activity, the participants were categorized into meal types according to the protein intake amount and composition in each meal. For example, in intake amount grouping, if participants consumed 20% of the daily protein intake during breakfast, 30% during lunch, and 50% during dinner, they were classified into the “dinner” group. On the other hand, for the composition type of protein intake, if participants consumed 20% of their energy during breakfast, 40% during lunch, and 30% during dinner, they were classified into the “lunch” group. The characteristics of each group are shown in Multimedia Appendix 2 and Multimedia Appendix 3. Moreover, weight, height, and frequency of recording were included in the dietary records, and these were also used in the multivariate logistic regression analysis.

### Physical Activity

The IPAQ short form assessed the amount of physical activity in daily life [24]. Respondents were asked about the number of days and hours spent doing each of the following 3 types of activities during the last 7 days: vigorous-intensity activity, moderate-intensity activity, and walking. Based on IPAQ analysis guidelines [25], we calculated weekly physical activity (metabolic equivalents-minutes/week [MET-min/week]) using the following formulas:

Walking MET-min/week = 3.3 × walking min × walking days **(4)**

Moderate MET-min/week = 4.0 × moderate-intensity activity min × moderate-intensity days **(5)**

Vigorous MET-min/week = 8.0 × vigorous-intensity activity min × vigorous-intensity days **(6)**

Total physical activity MET-min/week = sum of walking + moderate + vigorous MET-min/week values **(7)**

Data cleaning was performed according to previous guidelines [25]. These guidelines describe how to analyze data from the short and long versions of the IPAQ, which were obtained through interviews (telephone or interview) and self-administered questionnaires, and describe the procedures and data processing rules for both the short and long versions. The data processing included recoding of minimum values and truncation of data to improve data comparability.

### Statistical Analysis

Correlation analysis was conducted using Spearman rank correlation analysis. Moreover, multivariate logistic regression analysis was performed to investigate more detailed associations between dietary patterns and physical activity. A minimum sample size of 351 was required to have approximately 95% power to detect large effects at a significance level of .05 (G*Power, version 3.1.9.2; Universitat Kiel) in the multivariate logistic regression analysis. The objective variable, physical activity per exercise intensity, was divided into the following 2 groups: inactive (below median) and active (above median). We then divided the participants into groups according to their protein intake patterns and analyzed the amount of physical activity for each group using the Mann-Whitney *U* test. These analyses were conducted considering the following 2 factors: intake and composition. All data were analyzed using SPSS version 27 (IBM Corp), and a *P* value <.05 was considered to indicate statistical significance. Data are expressed as mean (SD) for most variables and as median and quartile (Q; 1-3) for IPAQ data.

## Results

### Participant Characteristics

From the obtained dietary records and questionnaires (19,319 responses), we excluded subjects for various reasons, and we eventually used the data of 8458 subjects (2321 male subjects and 6137 female subjects) in the present analysis (Figure 1). In particular, to focus on those who consume breakfast every day, we excluded about 5000 respondents, who made up the bulk of the excluded population. As this app has a gender gap among users, female individuals accounted for 73% of all participants in this analysis.

The mean participant age was 44.3 (SD 12.7) years, and the BMI was 25.0 (SD 3.7) kg/m^2^ for males and 22.9 (SD 3.9) kg/m^2^ for females (Table 1). The total energy intake was 2084.8 (SD 320.5) kcal for males and 1637.8 (SD 262.5) kcal for females, and the amount of energy per meal was skewed toward dinner. The daily protein intake was 85.5 (SD 17.2) g in males and 69.3 (SD 14.3) g in females, which exceeded the recommended amount of protein per day for Japanese people, as proposed by the Ministry of Health, Labor and Welfare in Japan [5]. Conversely, we observed that protein distribution was low during breakfast and skewed toward dinner, which is consistent with the findings of previous studies [6,7]. Indeed, there were significant differences in energy and protein intake comparing the 3 meals (energy: *P*<.001, protein: *P*<.001). In addition, the median amount of physical activity was 1257 (Q1-Q3 558-2346) MET-min/week. According to the Ministry of Health, Labor, and Welfare in Japan, the standard for physical activity for individuals aged 18 to 64 years is 23 MET-h/week, that is, 1380 MET-min/week of physical activity at an intensity of ≥3 MET [26]. Therefore, the distribution of the amount of physical activity in the current participants was slightly below the established standard.

**Table 1:** Characteristics of the participants.

Characteristic	Total (N=8458)	Male (n=2321)	Female (n=6137)
Age (years), mean (SD)	44.3 (12.7)	49.6 (11.4)	42.2 (12.5)
BMI (kg/m^2^), mean (SD)	23.5 (4.0)	25.0 (3.7)	22.9 (3.9)
Total energy (kcal/d), mean (SD)	1760.5 (343.5)	2084.8 (320.5)	1637.8 (262.5)
Breakfast energy (kcal), mean (SD)	389.6 (119.7)^a^	440.8 (129.8)^a^	370.2 (109.5)^a^
Lunch energy (kcal), mean (SD)	542.2 (128.7)	630.3 (133.1)	508.8 (109.9)
Dinner energy (kcal), mean (SD)	609.1 (191.6)	780.8 (185.6)	544.2 (148.9)
Total protein intake (g/d), mean (SD)	73.8 (16.8)	85.5 (17.2)	69.3 (14.3)
Breakfast protein (g/d), mean (SD)	16.9 (6.8)^a^	19.1 (7.4)^a^	16.1 (6.4)^a^
Lunch protein (g/d), mean (SD)	22.3 (6.2)	25.2 (6.5)	21.2 (5.7)
Dinner protein (g/d), mean (SD)	28.2 (8.0)	34.1 (8.0)	25.9 (6.7)
Total fat intake (g/d), mean (SD)	53.1 (13.1)	62.7 (12.9)	49.4 (11.2)
Breakfast fat (g/d), mean (SD)	12.3 (5.2)^a^	13.9 (5.6)^a^	11.7 (4.9)^a^
Lunch fat (g/d), mean (SD)	18.4 (5.8)	21.1 (6.2)	17.3 (5.2)
Dinner fat (g/d), mean (SD)	22.4 (7.6)	27.7 (7.5)	20.4 (6.6)
Total carbohydrate intake (g/d), mean (SD)	185.9 (44.5)	219.0 (46.1)	173.4 (36.9)
Breakfast carbohydrate (g/d), mean (SD)	53.0 (18.0)^a^	59.5 (20.1)^a^	50.6 (16.5)^a^
Lunch carbohydrate (g/d), mean (SD)	69.4 (18.2)	81.0 (19.3)	65.1 (15.7)
Dinner carbohydrate (g/d), mean (SD)	63.4 (22.7)	78.5 (24.0)	57.7 (19.2)
Total PA^b^ (MET^c^-min/week), median (Q1-Q3)	1257 (558-2346)	1470 (720-2612)	1164 (495-2232)
Vigorous PA (MET-min/week), median (Q1-Q3)	120 (0-960)	400 (0-1200)	0 (0-800)
Moderate PA (MET-min/week), median (Q1-Q3)	0 (0-360)	0 (0-360)	0 (0-360)
Walking (MET-min/week), median (Q1-Q3)	495 (198-990)	594 (198-1188)	495 (198-990)

^a^*P*<.001 by the Kruskal-Wallis test between breakfast, lunch, and dinner for energy and 3 macronutrients.

^b^PA: physical activity.

^c^MET: metabolic equivalents.

### Relationship Between Intake of the 3 Macronutrients and Physical Activity

Based on correlation analysis, each physical activity level had the strongest positive association with breakfast or lunch protein intake among the 3 macronutrients during the 3 meals (Table 2). Total, vigorous, and moderate physical activities were positively related to breakfast protein intake, while walking was positively related to lunch protein intake (total physical activity: *P*<.001, vigorous physical activity: *P*<.001, moderate physical activity: *P*<.001, walking: *P*<.001).

**Table 2:** Correlation analysis (Spearman rank correlation coefficient) of intake and composition of the 3 macronutrients and International Physical Activity Questionnaire findings.

Variable		Total PA^a^ (MET^b^- min/week)	Vigorous PA (MET-min/week)	Moderate PA (MET-min/week)	Walking (MET-min/week)
**Intake (g)**					
	Breakfast protein	0.170^c^	0.191^c^	0.072^c^	0.052^c^
	Breakfast fat	0.038^c^	0.021	0.019	0.033^d^
	Breakfast carbohydrate	0.041^c^	0.002	0.014	0.050^c^
	Lunch protein	0.142^c^	0.170^c^	0.039^c^	0.053^c^
	Lunch fat	0.016	0.0211	−0.009	0.017
	Lunch carbohydrate	0.023^e^	−0.001	−0.014	0.050^c^
	Dinner protein	0.091^c^	0.123^c^	0.011	0.037^c^
	Dinner fat	−0.014	0.010	−0.027^e^	−0.010
	Dinner carbohydrate	−0.011	−0.021	−0.030^d^	0.015
**Composition (% kcal)**					
	Breakfast protein	0.149^c^	0.196^c^	0.066^c^	0.023^e^
	Breakfast fat	−0.032^d^	−0.031^d^	−0.009	−0.006
	Breakfast carbohydrate	−0.047^c^	−0.086^c^	−0.022^e^	0.013
	Lunch protein	0.134^c^	0.175^c^	0.065^c^	0.023^e^
	Lunch fat	−0.021	−0.007	−0.004	−0.022^e^
	Lunch carbohydrate	−0.047^c^	−0.083^c^	−0.020	0.007
	Dinner protein	0.108^c^	0.134^c^	0.055^c^	0.028^e^
	Dinner fat	−0.055^c^	−0.024^e^	−0.012	−0.052^c^
	Dinner carbohydrate	−0.030^d^	−0.060^c^	−0.017	0.004

^a^PA: physical activity.

^b^MET: metabolic equivalents.

^c^*P*<.001 (Spearman rank correlation coefficient).

^d^*P*<.01 (Spearman rank correlation coefficient).

^e^*P*<.05 (Spearman rank correlation coefficient).

Multivariate logistic regression analysis revealed that vigorous and moderate physical activities were most strongly positively related with breakfast protein intake, even after adjustment for confounding factors, such as sex, age, BMI, and frequency of recording (Multimedia Appendix 4). On the other hand, although total physical activity was associated with breakfast protein intake, it showed the strongest positive relationship with lunch protein intake. Walking had a positive relationship with dinner protein intake.

### Relationship Between the Composition of the 3 Macronutrients (% kcal) and Physical Activity

Correlation analysis showed that total, vigorous, and moderate physical activities were positively associated with breakfast protein intake, while walking was positively associated with dinner protein intake (total physical activity: *P*<.001, vigorous physical activity: *P*<.001, moderate physical activity: *P*<.001, walking: *P*<.010) (Table 2). On the other hand, multivariate logistic regression analysis confirmed the strongest positive association of total physical activity with breakfast, and vigorous physical activity with lunch. No association with any of the nutrients could be confirmed for moderate exercise.

### Grouping by Protein Intake and Composition

The total protein intake per meal per day was similar between groups. Grouping by protein intake revealed that the breakfast and lunch groups demonstrated significantly greater physical activity (ie, total and vigorous physical activities) when compared with the dinner group (Table 3). In terms of walking, the lunch group presented significantly greater physical activity than the dinner group.

**Table 3:** International Physical Activity Questionnaire findings by meal type with the highest protein intake and composition from each meal.

Variable		Breakfast	Lunch		Dinner		*P* value^a^					
							B vs L^b^		B vs D^c^		L vs D^d^	
**Meal type with the highest protein intake**												
	Total PA^e^ (MET^f^-min/week), median (Q1-Q3)	1440 (693-2520)		1362 (597-2672)		1200 (528-2280)		.58		<.001		.001
	Vigorous PA (MET-min/week), median (Q1-Q3)	400 (0-1120)		240 (0-960)		80 (0-960)		.20		<.001		.02
	Moderate PA (MET-min/week), median (Q1-Q3)	0 (0-480)		0 (0-360)		0 (0-360)		>.99		.33		.95
	Walking (MET-min/week), median (Q1-Q3)	495 (198-990)		528 (198-1134)		495 (198-990)		>.99		>.99		.049
**Meal type with the highest protein composition**												
	Total PA (MET-min/week), median (Q1-Q3)	1386 (671-2597)		1266 (528-2376)		1188 (516-2234)		.02		<.001		.34
	Vigorous PA (MET-min/week), median (Q1-Q3)	400 (0-1120)		80 (0-960)		0 (0-840)		<.001		<.001		.04
	Moderate PA (MET-min/week), median (Q1-Q3)	0 (0-420)		0 (0-360)		0 (0-360)		.12		.02		>.99
	Walking (MET-min/week), median (Q1-Q3)	495 (198-990)		495 (198-990)		495 (198-990)		>.99		.94		>.99

^a^Mann-Whitney *U* test.

^b^Breakfast versus lunch.

^c^Breakfast versus dinner.

^d^Lunch versus dinner.

^e^PA: physical activity.

^f^MET: metabolic equivalents.

In addition, on grouping by protein composition, the breakfast group showed significantly higher total and vigorous physical activities than the lunch and dinner groups. Considering vigorous physical activity, the lunch group showed significantly greater physical activity than the dinner group. Finally, in terms of moderate physical activity, the breakfast group exhibited significantly greater physical activity than the dinner group.

## Discussion

### Principal Findings

In this study, we investigated the relationship of protein intake and composition during 3 meals with physical activity among adult male and female users of the mobile health app “Asken.” This study revealed that the daily amount of protein was higher than the recommended amount and the amount of physical activity was lower than the recommended amount. These findings could be attributed to the nature of the “Asken” app, which is a health care app typically designed to maintain or lose weight. In addition, because female individuals are generally more physically inactive than male individuals [27], the gender ratio of participants in this study may have influenced the results. Moreover, our results revealed that protein intake at breakfast and lunch showed the strongest positive relationship with physical activity among the 3 macronutrients consumed during the 3 meals. In addition, on grouping by protein intake patterns, those who consumed the most protein at breakfast and lunch exhibited significantly higher physical activity levels than those who consumed more protein at dinner. These results were similar for both intake and composition, indicating that not only intake but also the amount of protein in the overall diet is positively associated with the magnitude of physical activity. Several previous studies have reported that breakfast and lunch protein intakes affected the maintenance of muscle function [16,28,29]. Indeed, skeletal muscle mass increased in older individuals when protein was supplemented at breakfast and lunch [29]. A recent study has reported that in rodents, breakfast protein was effective in inducing muscle hypertrophy [16]. In addition, increased muscle mass has been shown to be negatively associated with mental health risks, such as depressed mood and stress, in addition to physical health [30,31]. Therefore, increasing protein intake at breakfast and lunch may enhance muscle mass, as well as promote physical and mental health. Indeed, among adolescent males and females, those with lower protein intake, especially those with lower milk intake at breakfast, have been reported to have lower scores for depression and anxiety symptoms [32]. Another possibility is that individuals with higher physical activity levels are more health conscious and typically consume more protein in the morning and afternoon. Several previous studies have reported a positive association between protein intake and physical activity levels [33-35]. However, these reports focused on daily protein intake, and few studies have examined intake at each meal. In addition, studies that have investigated protein intake in each diet have only investigated intake and have not confirmed the characteristics with respect to the distribution of intake [36]. Therefore, the finding that breakfast and lunch protein intakes are particularly related to physical activity is a new finding first presented in this study. Recently, 2 interesting longitudinal studies reported that high-quality protein evaluated by the “protein digestibility–corrected amino acid score” at breakfast but not lunch and dinner was positively associated with maintaining not only muscle strength but also brain cognition in Japanese older adults [37,38]. These studies have suggested future analysis of protein sources at breakfast using the “Asken” app.

### Study Strengths and Limitations

In this study, we investigated the relationship between the 3 major nutrients during the 3 traditional meals and physical activity using a large data set. In addition, we examined the characteristics of physical activity by grouping individuals according to the meal with the highest protein consumption. To the best of our knowledge, no previous study has examined dietary patterns of the 3 meals and daily physical activity in such a large data set. Accordingly, the strength of this study is that it analyzed the characteristics of physical activity according to daily dietary patterns, along with the relationship between protein intake and physical activity, which is an effective factor for the regulation of muscle function that has not been addressed in previous studies.

However, this study had several limitations. First, as this survey was based on self-reports by the participants, there may be discrepancies in their actual dietary data and physical activity levels. In other words, participants may have responded by exaggerating or underestimating their lifestyles based on social expectations. Second, given the nature of the app used in this study, most participants were female, and accordingly, menstruation could not be considered. Although the survey was analyzed by eliminating the influence of gender as appropriate, it could have marginally impacted the results. Third, the app offered courses for various health purposes, such as weight maintenance, weight loss, and muscle gain. Since we recruited subjects from among all users, there may have been a mix of subjects with different objectives, which may have affected the results. Fourth, this study did not take into account stress or sleep, which could have affected the quantity and content of the meals. That is, participants may have increased consumption of foods high in fat and carbohydrates. Fifth, our results showed a small value for the correlation coefficient. This may be partly due to the large sample size, but since a similar sample size was used in a previous study [39,40], we followed the same approach in our analysis. In addition, Spearman rank correlation analysis was conducted in this study to investigate the trend of association, and more detailed relationships were confirmed by multivariate logistic regression analysis. Finally, in the present survey experiments, we could not obtain timing information on snacks. Protein content (16.9 g) and percentage energy of protein (19.1%) may participate in the increase of physical activity when a snack is taken between breakfast and lunch.

### Conclusions

We analyzed the relationship of protein intake and composition during the 3 traditional meals with the amount of physical activity, as well as the characteristics of physical activity according to protein intake patterns in adult males and females. These results revealed that protein intake at breakfast and lunch had the strongest positive association with daily physical activity, and they were closely related to each other. The results suggest that breakfast may have an important role in physical activity. However, since this study focused only on protein, future studies should also consider protein sources.

## Appendix

Multimedia Appendix 1 Comparison of average nutrient intake between the National Nutrition Survey in Japan (NNSJ) 2019 and the data of this study.

Multimedia Appendix 2 Characteristics of meal types with the highest protein intake from each meal.

Multimedia Appendix 3 Characteristics of meal types with the highest protein composition from each meal.

Multimedia Appendix 4 Association of the intake and composition of the 3 macronutrients in each meal with International Physical Activity Questionnaire findings.

## Abbreviations

IPAQ: International Physical Activity Questionnaire
MET: metabolic equivalents
NNSJ: National Nutrition Survey in Japan
