# Correction: Relationship Between Protein Intake in Each Traditional Meal and Physical Activity: Cross-sectional Study

**DOI:** 10.2196/41460

**Published:** 2022-08-04

**Authors:** Takae Shinto, Saneyuki Makino, Yu Tahara, Lyie Nitta, Mai Kuwahara, Ayako Tada, Nanako Abe, Mikiko Michie, Shigenobu Shibata

**Affiliations:** 1 Department of Bioscience and Engineering Waseda University Tokyo Japan; 2 Asken Inc Tokyo Japan

In “Relationship Between Protein Intake in Each Traditional Meal and Physical Activity: Cross-sectional Study” (JMIR Public Health Surveill 2022;8(7):e35898), four errors were noted.

1. Due to a system error, in the originally published web version of the article, the name of one author, Lyie Nitta, was replaced with the name of another author, Mai Kuwahara. The order of authors appeared as follows:

Takae Shinto, Saneyuki Makino, Yu Tahara, Mai Kuwahara, Mai Kuwahara, Ayako Tada, Nanako Abe, Mikiko Michie, Shigenobu Shibata

This has been corrected to:

Takae Shinto, Saneyuki Makino, Yu Tahara, Lyie Nitta, Mai Kuwahara, Ayako Tada, Nanako Abe, Mikiko Michie, Shigenobu Shibata

2. In the originally published PDF version of the article, author Lyie Nitta's name appeared incorrectly as Lie Nitta. It has now been corrected in the article.

3. In the originally published web version of the article, author Mai Kuwahara was incorrectly linked to author Lyie Nitta’s ORCID iD. This discrepancy has now been corrected in the article.

4. In the originally published article, the copyright statement erroneously appeared as follows:

©Takae Shinto, Saneyuki Makino, Yu Tahara, Mai Kuwahara, Mai Kuwahara, Ayako Tada, Nanako Abe, Mikiko Michie, Shigenobu Shibata.

This has been corrected to:

©Takae Shinto, Saneyuki Makino, Yu Tahara, Lyie Nitta, Mai Kuwahara, Ayako Tada, Nanako Abe, Mikiko Michie, Shigenobu Shibata.

The correction will appear in the online version of the paper on the JMIR Publications website on August 4, 2022, together with the publication of this correction notice. Because this was made after submission to PubMed, PubMed Central, and other full-text repositories, the corrected article has also been resubmitted to those repositories.

