# Supplementary material for: Relationship Between Protein Intake in Each Traditional Meal and Physical Activity: Cross-sectional Study
Source: JMIR Public Health Surveill. 2022 Jul 12;8(7):e35898. doi: 10.2196/35898 (PMC9328787; doi:10.2196/35898)

Multimedia Appendix 1 Comparison of average nutrient intake between the National Nutrition Survey in Japan (NNSJ) 2019 and the data of this study

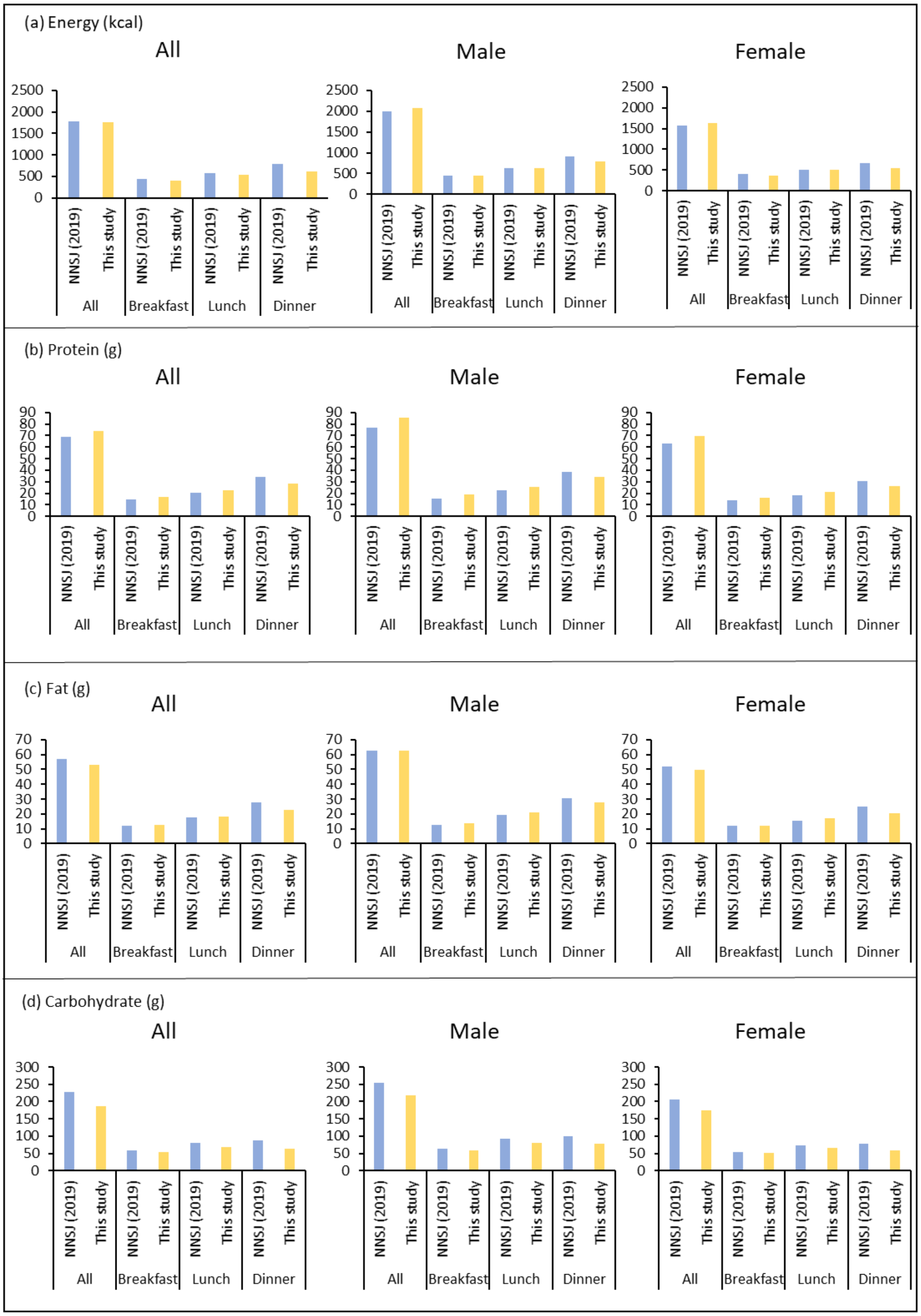

Supplement: Multimedia Appendix 1 [file publichealth_v8i7e35898_app1.pdf]
