# Supplementary material for: Relationship Between Protein Intake in Each Traditional Meal and Physical Activity: Cross-sectional Study
Source: JMIR Public Health Surveill. 2022 Jul 12;8(7):e35898. doi: 10.2196/35898 (PMC9328787; doi:10.2196/35898)
Supplement: Multimedia Appendix 2 [file publichealth_v8i7e35898_app2.pdf]

Multimedia Appendix 2 Characteristics of meal types with the highest protein intake from each meal.

**Male**	All (N=2321)		Breakfast (N=92)		Lunch (N=253)		Dinner (N=1976)	
	Mean	SD	Mean	SD	Mean	SD	Mean	SD
Total_energy (kcal/d)	2084.8	320.5	2023.1	288.7	2010.4	352.9	2097.2	316.0
Breakfast_energy (kcal/d)	440.8	129.8	538.2	138.1	445.2	132.0	435.7	127.3
Lunch_energy (kcal/d)	630.3	133.1	582.6	108.5	709.5	139.9	622.4	129.7
Dinner_energy (kcal/d)	780.8	185.6	631.7	146.0	621.1	178.6	808.2	174.2
Total_protein intake (g/d)	85.5	17.2	90.5	15.9	84.1	18.0	85.4	17.1
Breakfast_protein (g/d)	19.1	7.4	31.9	5.8	19.6	7.1	18.4	6.9
Lunch_protein (g/d)	25.2	6.5	24.2	6.2	31.7	6.1	24.4	6.1
Dinner_protein (g/d)	34.1	8.0	26.4	5.4	26.2	6.7	35.4	7.4
Total_fat intake (g/d)	62.7	12.9	59.3	12.8	60.3	13.9	63.2	12.7
Breakfast_fat (g/d)	13.9	5.6	17.5	6.1	14.1	5.7	13.7	5.5
Lunch_fat (g/d)	21.1	6.2	20.0	5.9	24.4	6.8	20.7	6.0
Dinner_fat (g/d)	27.7	7.5	21.8	6.1	21.8	7.3	28.8	7.2
Total_carbohydrate intake (g/d)	219.0	46.1	203.7	50.9	212.1	50.9	220.6	45.0
Breakfast_carbohydrate (g/d)	59.5	20.1	64.0	23.9	59.9	21.1	59.2	19.7
Lunch_carbohydrate (g/d)	81.0	19.3	72.7	18.2	86.1	22.6	80.7	18.7
Dinner_carbohydrate (g/d)	78.5	24.0	67.1	23.1	66.1	24.7	80.7	23.3
**Female**	All (N=6137)		Breakfast (N=419)		Lunch (N=1295)		Dinner (N=4423)	
	Mean	SD	Mean	SD	Mean	SD	Mean	SD

Total_energy (kcal/d)	1637.8	262.5	1587.7	281.0	1587.7	257.3	1657.2	259.7
Breakfast_energy (kcal/d)	370.2	109.5	486.7	119.8	365.4	107.5	360.6	102.6
Lunch_energy (kcal/d)	508.8	109.9	466.4	101.0	571.3	110.9	494.6	103.4
Dinner_energy (kcal/d)	544.2	148.9	419.8	132.9	436.2	127.0	587.6	132.7
Total_protein intake (g/d)	69.3	14.3	74.0	17.0	69.5	15.2	68.9	13.7
Breakfast_protein (g/d)	16.1	6.4	26.8	6.4	16.2	6.0	15.0	5.5
Lunch_protein (g/d)	21.2	5.7	20.1	5.5	26.0	5.4	19.9	5.0
Dinner_protein (g/d)	25.9	6.7	20.1	6.3	20.7	5.7	28.0	5.9
Total_fat intake (g/d)	49.4	11.2	46.0	11.6	47.8	11.2	50.2	11.0
Breakfast_fat (g/d)	11.7	4.9	15.6	5.7	11.7	4.9	11.4	4.6
Lunch_fat (g/d)	17.3	5.2	15.5	5.1	20.3	5.5	16.6	4.8
Dinner_fat (g/d)	20.4	6.6	14.9	5.9	15.8	5.6	22.2	6.0
Total_carbohydrate intake (g/d)	173.4	36.9	166.3	41.3	166.8	37.7	176.0	35.9
Breakfast_carbohydrate (g/d)	50.6	16.5	60.2	20.0	49.6	16.7	50.0	15.8
Lunch_carbohydrate (g/d)	65.1	15.7	59.6	15.7	69.3	17.3	64.4	14.9
Dinner_carbohydrate (g/d)	57.7	19.2	46.6	18.4	47.8	17.5	61.6	18.3
