# Supplementary material for: Relationship Between Protein Intake in Each Traditional Meal and Physical Activity: Cross-sectional Study
Source: JMIR Public Health Surveill. 2022 Jul 12;8(7):e35898. doi: 10.2196/35898 (PMC9328787; doi:10.2196/35898)
Supplement: Multimedia Appendix 3 [file publichealth_v8i7e35898_app3.pdf]

Multimedia Appendix 3 Characteristics of meal types with the highest protein composition from each meal.

**Male**	All (N=2321)		Breakfast (N=806)		Lunch (N=361)		Dinner (N=1154)	
	Mean	SD	Mean	SD	Mean	SD	Mean	SD
Total_energy (kcal/d)	2084.8	320.5	2111.3	321.3	2095.9	328.4	2062.9	316.0
Breakfast_energy (kcal/d)	440.8	129.8	431.3	136.2	441.4	137.3	447.2	122.2
Lunch_energy (kcal/d)	630.3	133.1	634.0	134.4	607.2	145.3	635.0	127.5
Dinner_energy (kcal/d)	780.8	185.6	806.9	180.3	821.9	199.4	749.8	179.6
Total_protein intake (g/d)	85.5	17.2	90.3	18.5	84.2	16.4	82.5	15.8
Breakfast_protein (% kcal)	17.8	6.8	22.9	8.3	15.1	3.8	15.0	3.6
Lunch_protein (% kcal)	16.2	3.7	16.3	3.6	19.1	5.2	15.2	2.7
Dinner_protein (% kcal)	17.9	4.3	17.1	4.2	16.2	3.7	19.1	4.3
Total_fat intake (g/d)	62.7	12.9	62.3	12.9	62.5	13.9	63.1	12.5
Breakfast_fat (% kcal)	28.2	7.8	28.1	7.6	28.4	9.0	28.2	7.5
Lunch_fat (% kcal)	29.9	5.7	30.0	5.9	30.2	6.3	29.9	5.3
Dinner_fat (% kcal)	32.3	6.4	31.0	6.4	31.0	7.0	33.6	6.0
Total_carbohydrate intake (g/d)	219.0	46.1	213.6	47.0	217.0	49.5	223.3	43.8
Breakfast_carbohydrate (% kcal)	53.9	10.0	49.1	9.9	55.9	10.2	56.5	8.8
Lunch_carbohydrate (% kcal)	51.5	7.2	50.9	7.3	48.7	8.0	52.7	6.5
Dinner_carbohydrate (% kcal)	40.6	9.9	39.9	10.5	40.5	11.3	41.1	9.0
**Female**	All (N=6137)		Breakfast (N=1419)		Lunch (N=779)		Dinner (N=3939)	
	Mean	SD	Mean	SD	Mean	SD	Mean	SD

Total_energy (kcal/d)	1637.8	262.5	1642.4	264.0	1636.8	274.7	1636.3	259.6
Breakfast_energy (kcal/d)	370.2	109.5	360.3	114.4	365.2	112.4	374.8	106.9
Lunch_energy (kcal/d)	508.8	109.9	508.5	111.4	492.4	118.1	512.2	107.4
Dinner_energy (kcal/d)	544.2	148.9	560.9	152.2	568.5	173.8	533.4	141.1
Total_protein intake (g/d)	69.3	14.3	74.4	16.0	67.9	14.2	67.8	13.3
Breakfast_protein (% kcal)	17.6	6.2	23.7	7.9	15.7	4.2	15.7	4.0
Lunch_protein (% kcal)	16.9	3.9	17.2	4.0	19.7	4.7	16.2	3.4
Dinner_protein (% kcal)	19.6	4.9	18.4	4.4	17.1	3.9	20.6	4.9
Total_fat intake (g/d)	49.4	11.2	48.9	11.2	48.9	11.9	49.7	11.0
Breakfast_fat (% kcal)	28.2	7.6	27.8	7.6	28.4	8.0	28.3	7.6
Lunch_fat (% kcal)	30.4	5.6	30.6	6.0	31.0	6.3	30.3	5.3
Dinner_fat (% kcal)	33.7	6.6	32.4	7.2	32.2	7.1	34.5	6.1
Total_carbohydrate intake (g/d)	173.4	36.9	168.9	37.8	171.5	39.5	175.3	35.9
Breakfast_carbohydrate (% kcal)	54.9	9.8	49.5	9.7	56.5	10.0	56.5	9.1
Lunch_carbohydrate (% kcal)	51.3	7.2	50.7	7.9	48.1	7.7	52.2	6.6
Dinner_carbohydrate (% kcal)	42.5	9.0	43.3	10.7	43.7	10.2	42.0	8.0
