# Supplementary material for: Relationship Between Protein Intake in Each Traditional Meal and Physical Activity: Cross-sectional Study
Source: JMIR Public Health Surveill. 2022 Jul 12;8(7):e35898. doi: 10.2196/35898 (PMC9328787; doi:10.2196/35898)
Supplement: Multimedia Appendix 4 [file publichealth_v8i7e35898_app4.pdf]

Multimedia Appendix 4 Association of the intake and composition of the 3 macronutrients in each meal with International Physical Activity Questionnaire findings.

	Total PA MET-min/week		Vigorous PA MET-min/week		Moderate PA MET-min/week		Walking MET-min/week	
	β	*P* value	*β*	*P* value	*β*	*P* value	*β*	*P* value
**Intake (g)**								
Breakfast_Protein	0.054	<0.001	0.089	<0.001	0.019	0.204	-0.008	0.604
Breakfast_Fat	0.011	0.449	-0.016	0.273	0.012	0.411	0.030	0.038
Breakfast_Carbohydrate	0.010	0.469	0.000	0.981	0.009	0.521	0.014	0.336
Lunch_Protein	0.078	<0.001	0.081	<0.001	0.039	0.029	0.034	0.052
Lunch_Fat	-0.030	0.076	-0.045	0.007	-0.017	0.328	0.004	0.830
Lunch_Carbohydrate	-0.018	0.236	-0.027	0.074	-0.018	0.245	0.008	0.611
Dinner_Protein	0.052	0.005	0.067	<0.001	-0.005	0.785	0.031	0.095
Dinner_Fat	-0.048	0.011	-0.041	0.031	-0.010	0.596	-0.042	0.029
Dinner_Carbohydrate	0.008	0.583	-0.011	0.462	0.011	0.445	0.020	0.188

**Composition (% kcal)**								
Breakfast_Protein	0.049	0.028	0.056	0.011	0.000	0.984	0.033	0.138
Breakfast_Fat	0.032	0.217	0.011	0.664	0.000	0.987	0.051	0.053
Breakfast_Carbohydrate	0.025	0.421	0.005	0.872	-0.009	0.763	0.050	0.106
Lunch_Protein	0.069	<0.001	0.078	<0.001	0.041	0.020	0.018	0.292
Lunch_Fat	0.012	0.580	0.004	0.841	0.008	0.722	0.013	0.551
Lunch_Carbohydrate	0.034	0.168	0.035	0.146	0.013	0.605	0.017	0.482
Dinner_Protein	0.027	0.052	0.040	0.003	0.002	0.909	0.007	0.640
Dinner_Fat	-0.040	0.004	-0.037	0.008	-0.012	0.407	-0.028	0.044
Dinner_Carbohydrate	-0.010	0.461	-0.024	0.079	0.005	0.710	0.003	0.825

Multivariable regression analyses adjusted by age, sex, BMI, and frequency of recording.
We divided the objective variable into 2 groups: below median defined as “0: inactive group”; over median defined as “1: active group”.

OR: odds ratio, CI: confidence intervals, R^2: adjusted coefficient of determination, logistic regression analyses adjusted by age, sex, BMI and frequency of recording
